# Supplementary material for: Low Expression of MATR3 Is Associated with Poor Survival in Clear Cell Renal Cell Carcinoma
Source: Biomedicines. 2023 Jan 24;11(2):326. doi: 10.3390/biomedicines11020326 (PMC9952985; doi:10.3390/biomedicines11020326)
Supplement: Supplementary file 1 [file biomedicines-11-00326-s001.zip › biomedicines-2107280-supplementary.pdf]

Supplementary data

Table S1. List of top 50 positively and 50 negatively correlated genes with MATR3 in ccRCC.

Positively Correlated Genes			Negatively Correlated Genes		
	**Genes**	**PearsonCC**		**Genes**	**PearsonCC**
1	SMAD5	0.92	1	C19orf53	-0.6
2	PJA2	0.9	2	SERF2	-0.56
3	PGGT1B	0.9	3	TAF10	-0.56
4	RBM27	0.9	4	POLR2J	-0.55
5	C11orf58	0.9	5	TRAPPC5	-0.55
6	PRKAA1	0.9	6	ZNHIT1	-0.54
7	ACAP2	0.89	7	YDJC	-0.51
8	BCLAF1	0.89	8	GADD45GIP1	-0.51
9	PHAX	0.89	9	COX6B1	-0.5
10	CTDSPL2	0.89	10	NDUFA3	-0.5
11	DEK	0.89	11	BLOC1S1	-0.5
12	GABPA	0.89	12	TIMM16	-0.49
13	CDC73	0.89	13	C17orf90	-0.49
14	TBC1D15	0.88	14	LSM7	-0.49
15	FAM114A2	0.88	15	FKBP2	-0.49
16	DIS3	0.88	16	DNAJC4	-0.49
17	SPOPL	0.88	17	TCEB2	-0.49
18	AGGF1	0.88	18	MRPL23	-0.48
19	SP3	0.88	19	NDUFS8	-0.48
20	UBE3A	0.88	20	ROMO1	-0.48
21	VPS26A	0.88	21	NME3	-0.48
22	NPAT	0.88	22	APRT	-0.47
23	SRFBP1	0.88	23	NCRNA00116	-0.47
24	NEDD1	0.88	24	C9orf142	-0.47
25	SLC25A24	0.87	25	MIF	-0.47
26	MARCH7	0.87	26	POLD4	-0.47
27	HIAT1	0.87	27	PPP1R14B	-0.46
28	CHM	0.87	28	C19orf24	-0.46
29	POLK	0.87	29	ZDHHC24	-0.46
30	PUM2	0.87	30	SSNA1	-0.46
31	WAPAL	0.87	31	KRTCAP2	-0.46
32	TTC33	0.87	32	RNPEPL1	-0.46
33	KHDRBS1	0.87	33	PUSL1	-0.46
34	PRRC1	0.87	34	DRAP1	-0.45
35	ATRX	0.87	35	SHARPIN	-0.45
36	C5orf24	0.87	36	HSPB1	-0.45
37	STAM2	0.87	37	RPS15	-0.45
38	USP1	0.87	38	MRPL41	-0.45
39	FAM98B	0.87	39	MXD3	-0.45
40	CNOT8	0.87	40	PGLS	-0.45
41	FBXL3	0.86	41	MRPS15	-0.45

42	ITCH	0.86	42	PSENEN	-0.45
43	STAG2	0.86	43	C16orf13	-0.45
44	NUS1	0.86	44	ROBLD3	-0.45
45	G3BP1	0.86	45	C7orf47	-0.45
46	API5	0.86	46	SMUG1	-0.45
47	RAB6A	0.86	47	HCFC1R1	-0.45
48	CNOT6	0.86	48	GPX4	-0.44
49	PPP6C	0.86	49	C17orf37	-0.44
50	STX12	0.86	50	NUTF2	-0.44

Table S2. Multivariate analysis of prognostic factors by the Cox proportional hazard model for MRPL23 expression and combination of MRPL23 and MATR3.

	**Multivariate analysis: MRPL23**				**Multivariate analysis: MATR3+MRPL23**			
**Variable**	**HR**	**95% CI**		**P-value**	**HR**	**95.0% CI**		**P-value**
**MRPL23**	1.94	1.41	2.67	**<0.0001**	-	-	-	-
**MATR3-h/MRPL23-l**	-	-	-	-	**Ref.**			
**MATR3-l/MRPL23-h**	-	-	-	-	3.15	2.05	4.83	**<0.0001**
**others**	-	-	-	-	2.11	1.44	3.11	**0.0001**
**Sex**	-	-	-	-	-	-	-	-
**Age**	-	-	-	-	-	-	-	-
**Grade**	1.16	0.84	1.61	0.37	1.14	0.83	1.59	0.42
**TNM stage**	3.10	2.21	4.35	**<0.0001**	3.22	2.30	4.51	**<0.0001**

Table S3. Clinical characteristics of 107 patients with ccRCC from our cohort.

**Variables**	Number (%)
**Sex**	
**Females**	32 (29.91)
**Males**	75 (70.09)
**Age**	
**≤64**	59 (55.14)
**>64**	48 (44.86)
**Grade**	
**G1**	26 (24.30)
**G2**	68 (63.55)
**G3**	12 (11.21)
**G4**	1 (0.93)
**pT status**	
**T1**	31 (28.97)
**T2**	30 (28.04)
**T3**	43 (40.19)
**T4**	3 (2.80)
**cN status**	
**N0**	100 (93.46)
**N1**	7 (6.54)

Table S4. Clinical characteristics of 475 patients with ccRCC from TCGA cohort.

Variables	Number (%)
**Sex**	
**Females**	163 (34.32)
**Males**	312 (65.68)
**Age**	
**≤60**	239 (50.32)
**>60**	236 (49.68)
**Grade**	
**G1**	11 (2.32)
**G2**	203 (42.74)
**G3**	189 (39.79)
**G4**	72 (15.16)
**pT status**	
**T1**	237 (49.89)
**T2**	61 (12.84)
**T3**	167 (35.16)
**T4**	10 (2.11)
**pN status**	
**Nx**	235 (49.47)
**N0**	225 (47.37)
**N1**	15 (3.16)
**Stage**	
**I**	234 (49.26)
**II**	50 (10.53)
**III**	119 (25.05)
**IV**	72 (15.16)

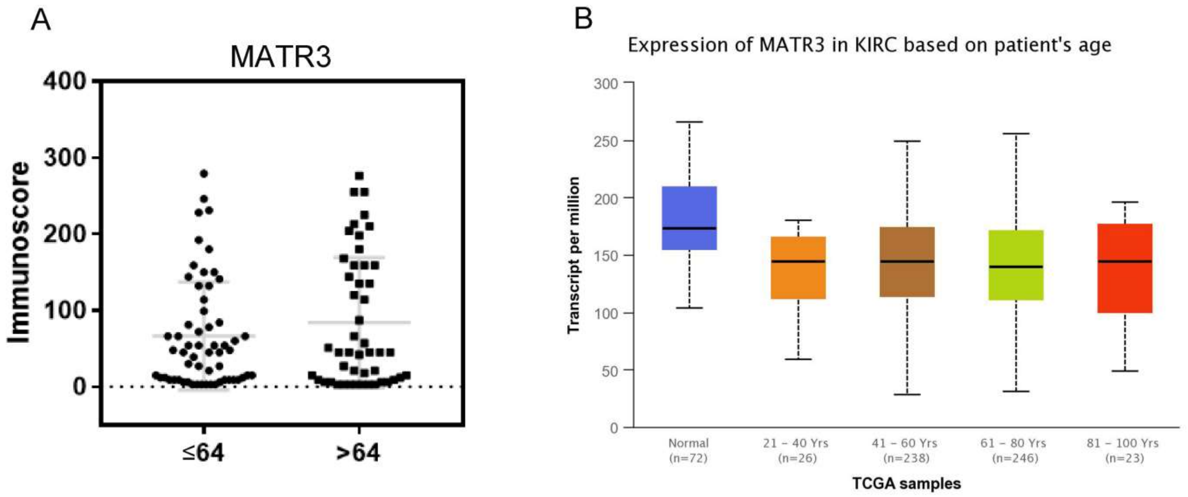

Figure S1. MATR3 expression depending on the age of patients. MATR3 expression depending on the age of patients in our cohort (A); and based on the UALCAN database (B). KIRC - Kidney Renal Clear Cell Carcinoma

DAVID database

A

Annotation Cluster 1	Enrichment Score: 4.52	G		Count	P_Value	Benjamini
UP_KW_MOLECULAR_FUNCTION	Ribosomal protein	RT		5	7.5E-8	1.5E-7
UP_KW_MOLECULAR_FUNCTION	Ribonucleoprotein	RT		5	4.7E-7	4.7E-7
REACTOME_PATHWAY	Translation	RT		5	5.0E-7	2.3E-5
REACTOME_PATHWAY	Mitochondrial translation initiation	RT		4	2.0E-6	2.3E-5
REACTOME_PATHWAY	Mitochondrial translation elongation	RT		4	2.0E-6	2.3E-5
REACTOME_PATHWAY	Mitochondrial translation termination	RT		4	2.0E-6	2.3E-5
REACTOME_PATHWAY	Mitochondrial translation	RT		4	2.4E-6	2.3E-5
GOTERM_CC_DIRECT	ribosome	RT		4	2.9E-6	5.2E-5
GOTERM_MF_DIRECT	structural constituent of ribosome	RT		4	4.5E-6	2.7E-5
GOTERM_BP_DIRECT	translation	RT		4	6.2E-6	8.6E-5
KEGG_PATHWAY	Ribosome	RT		3	3.7E-4	7.5E-4
UP_KW_CELLULAR_COMPONENT	Mitochondrion	RT		4	4.1E-4	8.1E-4
REACTOME_PATHWAY	Metabolism of proteins	RT		5	1.1E-3	8.5E-3
GOTERM_CC_DIRECT	mitochondrion	RT		4	1.3E-3	1.2E-2
GOTERM_MF_DIRECT	RNA binding	RT		4	1.7E-3	5.2E-3
GOTERM_CC_DIRECT	mitochondrial inner membrane	RT		3	2.8E-3	1.7E-2
GOTERM_MF_DIRECT	protein binding	RT		4	5.9E-1	1.0E0

B

Annotation Cluster 2	Enrichment Score: 1.67	G		Count	P_Value	Benjamini
GOTERM_BP_DIRECT	intracellular protein transport	RT		4	4.5E-6	8.6E-5
UP_KW_BIOLOGICAL_PROCESS	Protein transport	RT		3	3.3E-3	1.3E-2
UP_KW_BIOLOGICAL_PROCESS	Transport	RT		3	3.5E-2	7.1E-2
UP_KW_PTM	Acetylation	RT		3	1.5E-1	5.2E-1
UP_KW_PTM	Phosphoprotein	RT		4	2.1E-1	5.2E-1
GOTERM_MF_DIRECT	protein binding	RT		4	3.0E-1	1.0E0
UP_KW_CELLULAR_COMPONENT	Membrane	RT		3	4.5E-1	6.0E-1

Figure S2. The subnetwork functional enrichment analysis of MCODE cluster 1 (A) and cluster 2 (B) using the *DAVID Functional Annotation Clustering* Tool.

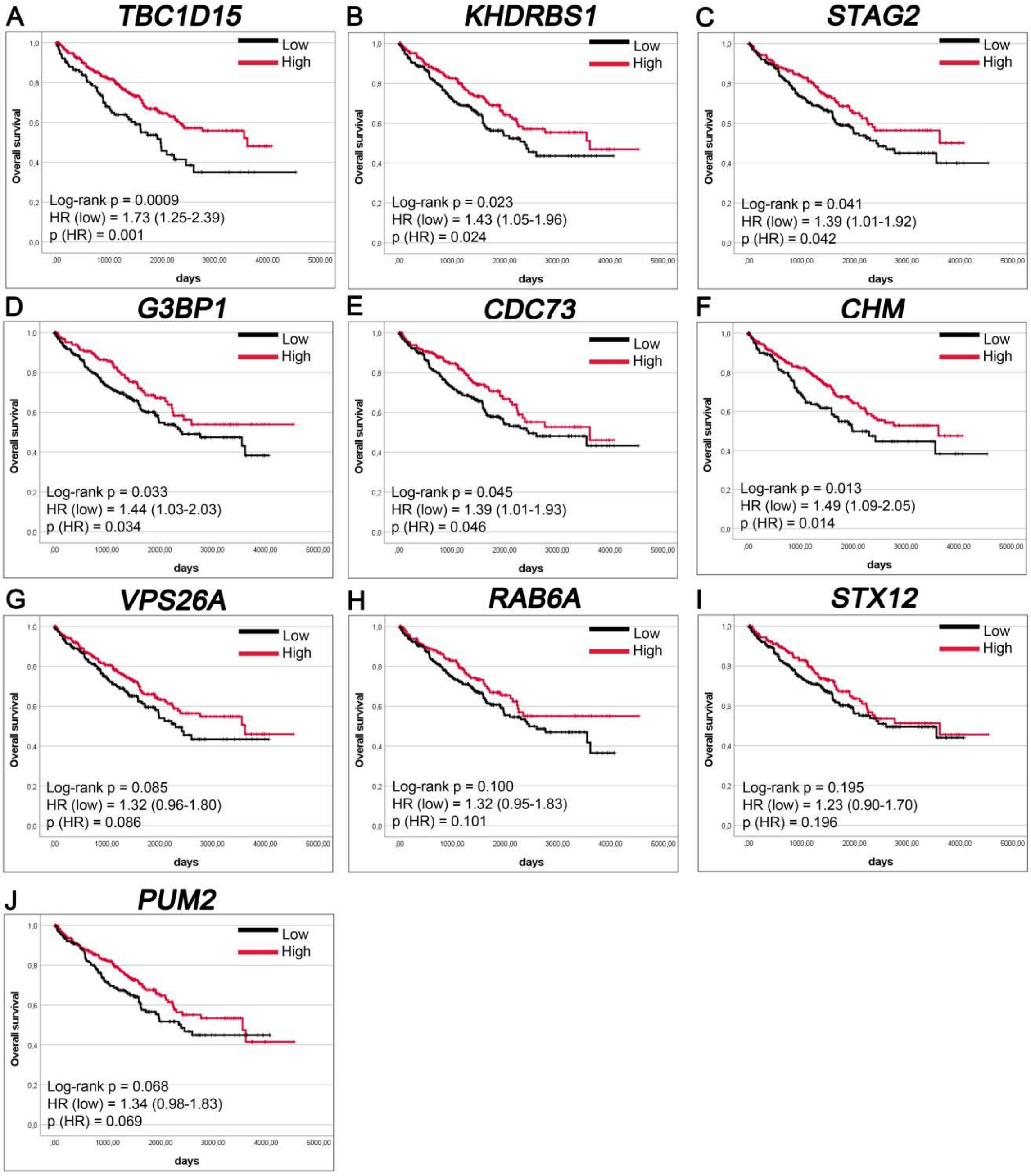

Figure S3. Kaplan–Meier curves for overall survival (OS) of clear cell renal cell carcinoma (ccRCC) patients stratified by *TBC1D15* (A), *KHDRBS1* (B),*STAG2* (C), *G3BP1* (D), *CDC73* (E), *CHM* (F), *VPS26A* (G), *RAB6A* (H), *STX12* (I), *PUM2* (J). The survival curves were plotted based on the TCGA ccRCC dataset sourced from the UCSC Xena database. Cases were divided into expression groups (low and high) according to the optimal cutoff point determined by the Evaluate Cutpoints software. The results are displayed with hazard ratio (HR) from the Cox PH model and Cox *p*-value or p-value from log-rank test.

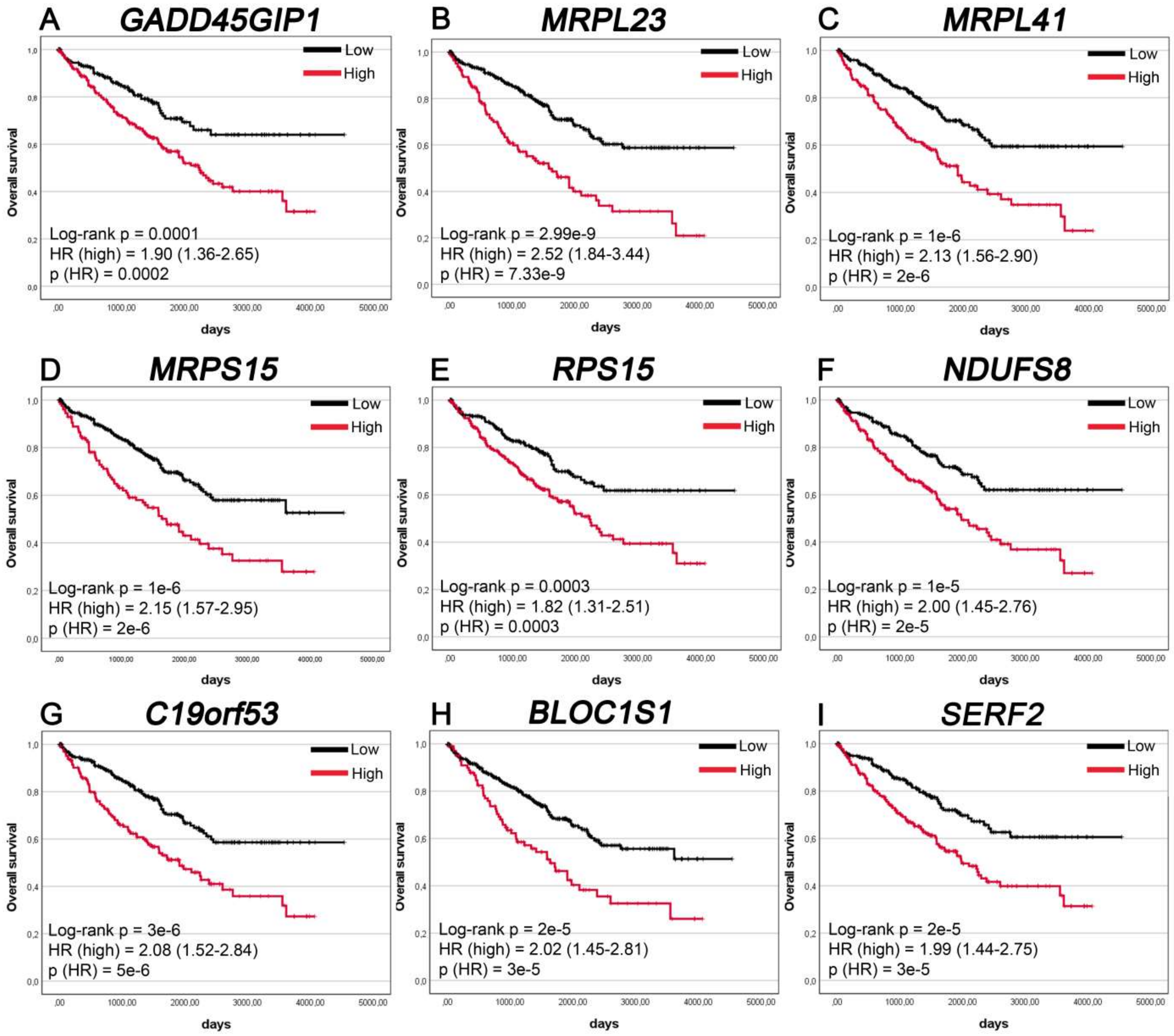

Figure S4. Kaplan–Meier curves for overall survival (OS) of clear cell renal cell carcinoma (ccRCC) patients stratified by *GADD45GIP1* (A), *MRPL23* (B), *MRPL41* (C), *MRPS15* (D), *RPS15* (E), *NDUFS8* (F), *C19orf53* (G), *BLOC1S1* (H), *SERF2* (I). The survival curves were plotted based on the TCGA ccRCC dataset sourced from the UCSC Xena database. Cases were divided into expression groups (low and high) according to the optimal cutoff point determined by the Evaluate Cutpoints software. The results are displayed with hazard ratio (HR) from the Cox PH model and Cox *p*-value or p-value from log-rank test.

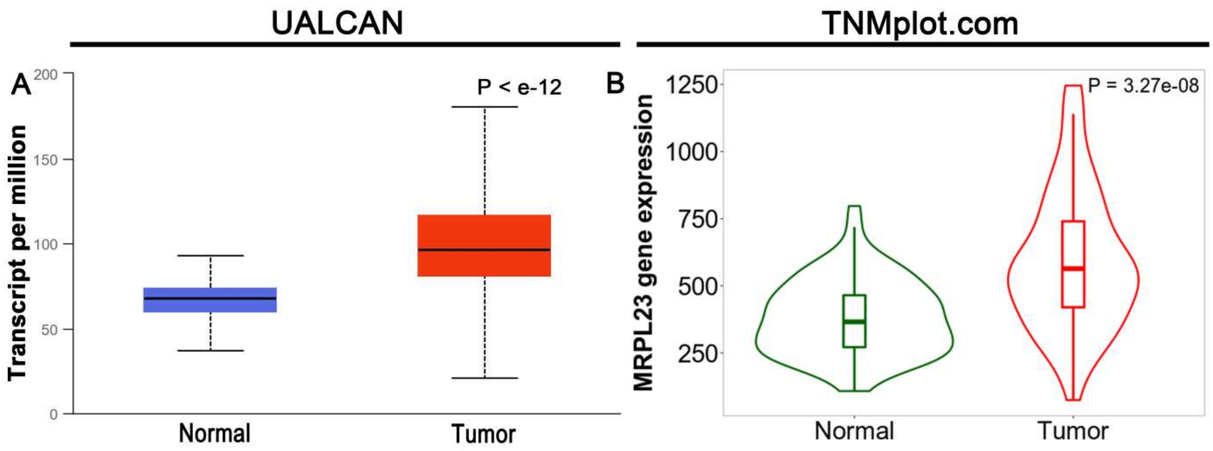

Figure S5. Differential expression of *MRPL23* in clear cell renal cell carcinoma **(**ccRCC) and normal tissues. Box plot visualized using UALCAN database (A) and violin plot visualized using TNMplot.com web tool (B) of *MRPL23* gene expression in ccRCC when compared to adjacent normal and tumor RNA-seq TCGA data.

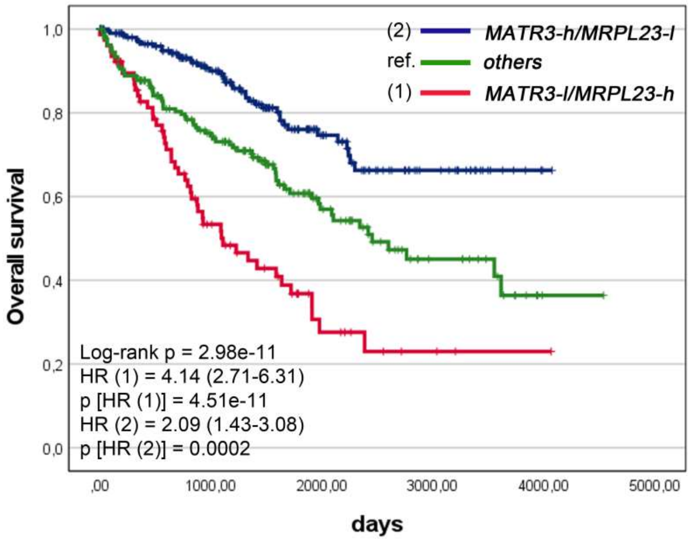

Figure S6. Kaplan–Meier curves for overall survival (OS) of clear cell renal cell carcinoma (ccRCC) patients stratified by combined expression of *MATR3* and *MRPL23*. The survival curves were plotted based on the TCGA ccRCC dataset sourced from the UCSC Xena database. The results are displayed with hazard ratio (HR) from the Cox PH model and Cox *p*-value or p-value from log-rank test.
